# Models of persecutory delusions: a mechanistic insight into the early stages of psychosis

**DOI:** 10.1038/s41380-019-0427-z

**Published:** 2019-05-10

**Authors:** Andreea Oliviana Diaconescu, Daniel Jonas Hauke, Stefan Borgwardt

**Affiliations:** 1 Department of Psychiatry (UPK), University of Basel, Basel, Switzerland; 2 Translational Neuromodeling Unit (TNU), Institute for Biomedical Engineering, University of Zurich & Swiss Federal Institute of Technology (ETH Zurich), Zurich, Switzerland; 3 Department of Mathematics and Computer Science, University of Basel, Basel, Switzerland; 4 Department of Psychosis Studies PO63, Institute of Psychiatry, Psychology and Neuroscience, King’s College London, London, UK

**Keywords:** Schizophrenia, Predictive markers

## Abstract

Identifying robust markers for predicting the onset of psychosis has been a key challenge for early detection research. Persecutory delusions are core symptoms of psychosis, and social cognition is particularly impaired in first-episode psychosis patients and individuals at risk for developing psychosis. Here, we propose new avenues for translation provided by hierarchical Bayesian models of behaviour and neuroimaging data applied in the context of social learning to target persecutory delusions. As it comprises a mechanistic model embedded in neurophysiology, the findings of this approach may shed light onto inference and neurobiological causes of transition to psychosis.

## Introduction

Persecutory delusions, defined as unfounded beliefs that others are deliberately intending to cause harm, are core symptoms of psychosis and a burden for patients [1]. Persecutory ideation leads to increased incidence of violent behaviour [2], suicidal ideation and relapse [3]. About half of the first-episode psychosis (FEP) patients with persecutory delusions show psychological well-being levels lower than 2% of the general population [4].

A recent approach to treatment of psychosis focuses on early detection and prevention. However, a fundamental problem for research on the early phases of psychosis is identifying robust markers for transition to psychosis from the clinical high-risk state (CHR) [5]. The CHR is defined by the presence of one or more of the following criteria: attenuated psychotic symptoms, brief limited intermittent psychotic episodes, trait vulnerability, as well as a marked decline in psychosocial functioning and unspecified prodromal symptoms. Whereas clinical variables have good prognostic accuracy for ruling out individuals who will not develop psychosis, there is a need to improve the prediction accuracy of future transition to psychosis [5, 6].

Previous studies have examined the predictive value provided by neuroimaging methods including structural [7–10] and functional magnetic resonance imaging (MRI) [11, 12]. In contrast to clinical and environmental variables, whole-brain examinations of structural MRI data using voxel-based morphometry delivered the largest prediction accuracy rates, reaching ~ 80% prediction accuracy in a cross-centre study [7]. A recent review of predictive models for psychosis transition indicated that using multiple variables (biological, environmental, and neurocognitive), and testing them sequentially in CHR individuals may substantially improve prediction rates [6]. This suggests that a multimodal, combinatorial approach is needed.

Although current methods link transition risk with particular differences in genetic polymorphisms or brain structures, they do not allow for quantifying the probability that a particular disease mechanism is present. This, however, is the basis for targeted treatment.

One solution for identifying disease mechanisms is to pursue a computational modelling strategy and employ generative models that focus on core symptoms, such as persecutory delusions. Generative models describe mechanisms that could have generated the observed behaviour or neuroimaging data. Individual differences in behaviour—potentially related to disease mechanisms—can be uncovered by estimating individual model parameters based on participants’ behaviour [13]. In addition to pure risk prediction, this approach, because it is mechanistic, may also prove useful for identifying pathophysiological mechanisms of emerging psychosis (see Supplementary Figure 1).

One class of generative models, which can be fit to noninvasive measurements (electroencephalogram (EEG) or functional magnetic resonance imaging (fMRI)), is models of effective connectivity such as dynamic causal modelling (DCM), describing causal (directed) influences between neurons or neuronal populations [14]. DCMs explain measured brain activity as arising from circuit dynamics that are a function of (i) intrinsic connectivity, (ii) experimentally induced perturbations, and (iii) modulatory inputs that invoke contextual changes in synaptic strengths (i.e., short-term plasticity during learning or neuromodulatory influences). A complementary approach to neuroimaging-based models is afforded by generative models of behaviour. These can be fitted to trial-by-trial behavioural responses to capture (mal)adaptive aspects of learning and decision-making [15].

Here, we introduce a computational framework that focuses on a central symptom of psychosis, namely persecutory ideation. This framework integrates computational models of behaviour with neural circuit models, which describe the neuronal causes of aberrant learning and can be fit to EEG and fMRI data. It makes specific predictions about pathophysiology in psychosis, which may be used to predict transition to psychosis in CHR individuals and treatment response in FEP patients.

## Computational accounts of persecutory delusions

Delusions in general are conceptualised as false beliefs based on incorrect inference about the external world, which persist in the face of disconfirmatory evidence. Two major computational theories exist, which assume specific mechanisms of delusional belief genesis and persistence.

First, a popular notion is that patients with psychosis attribute inappropriately high aberrant salience to irrelevant events. This theory posits a key role of the dopamine system in mediating the misattribution of salience (for a review, see ref. [16]). It is consistent with well-established theories of increased phasic dopamine release in psychosis [17–20] and supported by a host of fMRI studies in FEP patients [21–23]. Although compelling, this theory does not provide an explanation how aberrant salience attribution leads to the development of uncorrectable delusional beliefs.

A second and related theory of delusions focuses on the Bayesian brain hypothesis and the interplay between prior beliefs and “correction” signals or prediction errors (PEs) [24, 25]. The Bayesian account of perception proposes that the brain generates predictions about its sensory inputs and adjusts those predictions via incoming PEs [26, 27]. Adopting a hierarchical Bayesian framework, beliefs at multiple levels, from discrete sensory events to more abstract aspects of the environment (e.g., probabilistic associations and volatility), are updated based on precision-weighted PEs [28, 29]. Specifically, in hierarchical models, a ratio of precisions (assigned to sensory inputs relative to prior beliefs) serves to scale the amplitude of PE signals and thus their impact on belief updates [28].

**Fig. 1:**
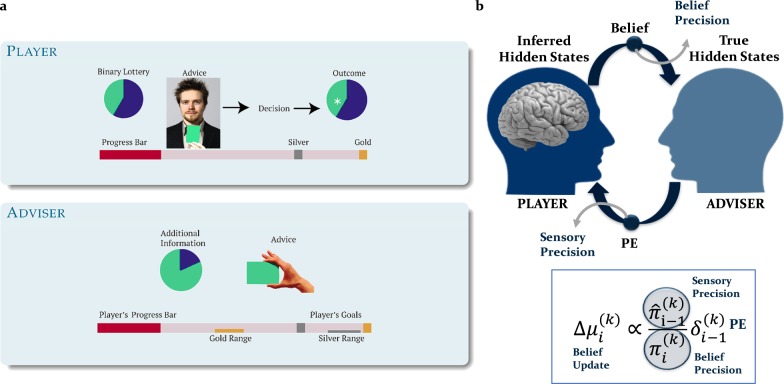
Probing persecutory ideation: inferring on others’ intentions experimental paradigm and computational model. **a** Participants took part in a face-to-face advice-taking task for monetary rewards and were randomly assigned to “player” and “adviser” roles. “Players” had to predict the outcome of a binary lottery draw, whereas “Advisers” gave Players suggestions on which option to choose. Both sets of participants received incentives and the pay-off structure differed to ensure the presence of both collaboration and competition between the two participants. Players profited from the Adviser’s recommendations as Advisers always received more information about the outcome of the lottery (constant probability of 80%), whereas Advisers gained from the Players’ compliance to take the advice into account. The Advisers’ motivation to provide valid or misleading information varied during the game as a function of their own incentive structure. Players were (truthfully) informed that the Adviser had his own (undisclosed) incentive structure and because of it, intentions could change during the game (volatility). The social learning task was adapted for fMRI or EEG recordings by using 2-sec video clips of the Advisers recorded during the interactive sessions. **b** According to the model, an agent infers on true hidden states in the world by continuously updating his/her predictions (or beliefs) via precision-weighted prediction errors (PEs). Assuming Gaussian distributions over beliefs, these can be described by their sufficient statistics, the mean (*μ*) and the variance/uncertainty (*σ*) or its inverse precision/certainty (*π*). Predictions about hidden states in the world (before observing an outcome) are denoted with a hat symbol (e.g.,$\hat \pi$). At each hierarchical level *i*, belief updates (updates of the posterior means $\mu _i^{(k)}$) on each trial *k* are proportional to precision-weighted PEs. The belief update is the product of the PE from the level below $\delta _{i - 1}^{(k)}$, weighted by a precision ratio. The ratio is composed of $\hat \pi _{i - 1}^{\left( k \right)}$ and $\pi _i^{\left( k \right)}$, which represent estimates of the precision of the predicted input from the level below (sensory precision) and precision of the belief at the current level, respectively

Recent theories of perceptual abnormalities in psychosis have built on hierarchical Bayesian frameworks, extending the concept of aberrant salience by highlighting the role of uncertainty (or its inverse, precision) [24, 30–33]. One specific suggestion from these accounts is that aberrantly strong (or precise) incoming PEs indicate that prior predictions are inadequate and beliefs or actions must be changed to accurately predict states in the world. Thus, a plethora of incoming error signals leads to a brittle (or uncertain) model about states in the world, which ultimately sets the stage for the formation of delusions [34, 35]. High-order beliefs of abnormally low precision lead to a lack of regularisation, which renders the environment seemingly unpredictable and volatile, enhancing the weight of incoming PEs [33]. A brittle model of the world may require adoption of extraordinary higher-order beliefs [32, 36]. Notably, these explanations are not exclusive but could co-exist; specifically, they relate to numerator and denominator of the precision ratio in Eq. 1 of Figure 1 (see Supplementary material for additional details).

Fully developed delusions could be understood as implausible beliefs with overly high precision, which function to attenuate aberrant sensory evidence [33]. Recent studies have shown that strong prior beliefs govern the belief-updating process in individuals who reported auditory hallucinations (hearing voices) [37]. Prior beliefs were also more resistant to change in psychosis patients with acute delusions [38]. Furthermore, the utilisation of prior knowledge correlated with positive symptom severity in a perceptual discrimination task [39]. However, the study also reported decreased impact of experimentally induced priors on the behaviour of psychosis patients [39] (also see ref. [40]). On the other hand, a recent study found that delusion-prone individuals showed a reduced influence of experimental priors in perceptual but not cognitive discrimination tasks [41]. These somewhat ambiguous results may be reconciled by a developmental change in prior utilisation and/or distinct impact of belief precision at different levels of the processing hierarchy [33, 36].

In the context of psychosis, the most-prominent delusional beliefs pertain to the social world and result from inference about the mental states of others, specifically that their intentions are of a persecutory nature [42, 43]. A precise predictive model is particularly important for social contexts when interpreting others’ intentions [44, 45], because human intentions are typically concealed or only expressed indirectly, requiring predictions from observations of ambiguous behaviour. Higher-level prior beliefs, which shape one’s perception of others, may arise from one’s own psychotic experiences including hearing voices, as individuals tend to regard their own predictions about states in the world as more reliable than second person accounts [46].

Computational models of persecutory delusions must be based on existing cognitive models. Key cognitive predispositions for persecutory ideation are in line with the hypothesis of an initially uncertain predictive model of others’ intentions (for a review, see ref. [42, 47–49]). Individuals who later develop persecutory delusions report high levels of worry and rumination about how others perceive them [50, 51]. These findings relate to the proposal of weak prior beliefs leading to causal misattribution [35]. The notion that persecutory ideation may be associated with abnormal inference and imprecise prior beliefs has been related to the Jumping to Conclusions bias (e.g., [52–54]; but see refs. [55] and [56] for alternative interpretations). Individuals with persecutory delusions may adopt implausible explanations in social contexts [38] and overly negative attributions about others (e.g., negative events are attributed to active, malevolent intentions of another person) [57].

With regard to pathophysiology, psychosis represents a spectrum of disturbances in the interaction between *N*-methyl-d-aspartic acid (NMDA)-receptor dependent synaptic plasticity and neuromodulatory systems like dopamine and acetylcholine (see ref. [58] for a review and [59, 60] for recent empirical findings). However, the link between impaired social cognition, persecutory delusions, and disruptions in synaptic plasticity by neuromodulatory systems has not been established. This is because it requires ecologically valid and deception-free experimental paradigms that have also been studied neurobiologically.

Here, we propose such a paradigm to test the hypothesised link between social inference and persecutory ideation. This paradigm was adapted from a previous social learning task [61] and probes how one infers on the intentions of another agent (adviser) who provides iterative advice about the outcome of a probabilistic task based on additional information that he/she obtains on every trial (Fig. 1a). Importantly, this task maps onto existing pathophysiological mechanisms of psychosis [62].

## Inferring on others’ intentions: a framework for probing persecutory delusions

To understand the genesis and persistence of persecutory delusions the computational framework needs to be examined in an experimental context that is sensitive to the process of interest. Therefore, we propose a paradigm that has been developed to specifically address persecutory ideation, as it requires learning about the hidden and possibly changing intentions of another person. It requires hierarchical processing from non-social to social representations with increasing levels of abstraction, which can be mapped onto hypothesised pathophysiological mechanisms of psychosis, in particular precision-weighted PE belief-updating [62, 63].

Participants perform a binary lottery task and are additionally given advice from a more informed agent (the adviser) about which option to choose. In order to perform well, they not only have to predict the accuracy of current advice, but also the adviser’s intention and how it might change over time (i.e., volatility) (Fig. 1a, upper panel). To examine the impact of precision on learning from advice, we manipulated volatility and thereby varied the association strength between the advice and the outcome. We assumed that the higher-level belief precision about the adviser’s fidelity is low, when volatility is high and vice-versa.

The adviser’s intentions and motivation to provide helpful advice change according to the incentive structure of the task (Fig. 1a, lower panel). The task was adapted for testing along with either EEG or fMRI recordings by replacing face-to-face interactions with videos of the advisers, taken from trials when advisers truly intended to help or to mislead the players [62, 63]. This ensured that all participants received the same input structure and therefore could be compared in terms of their learning parameters and how they inferred from advice. Although each participant received the same advice sequence throughout the task, the advisers displayed in the videos varied between participants to ensure that physical appearance and gender did not impact on their decisions to take advice into account.

While there are other multiround trust games, which could potentially be used to examine persecutory ideation (see ref. [57, 64]), there are several features of the current paradigm that make it particularly useful for probing persecutory ideation. First of all, it is ecologically valid: the videos of advice reflected instances when the adviser truly intended to help or truly intended to mislead the participant. Second, it is deception-free: participants were fully informed that the adviser had a different incentive structure and thus was motivated to not always offer helpful advice (see ref. [65] for details). Third, in contrast to other theory of mind (e.g. the mind in the eye task, emotion recognition tasks, variations of the Sally-Ann task) or decision-making tasks (a single-shot or short multiround dictator or trust game), this paradigm includes a prolonged, iterative interaction, which allows the examination of how beliefs are updated as a result of contradicting evidence or PEs. Fourth, it provides a context to test what we hypothesise to be impaired in persecutory ideation, namely the different contributions of sensory compared with belief precision. Finally, the paradigm includes volatility (owing to the incentive structure offered to advisers), which can be used to manipulate the players’ confidence about their estimates of adviser’s fidelity.

## Inferring on others’ intentions as precision-weighted PE updates

**Fig. 2:**
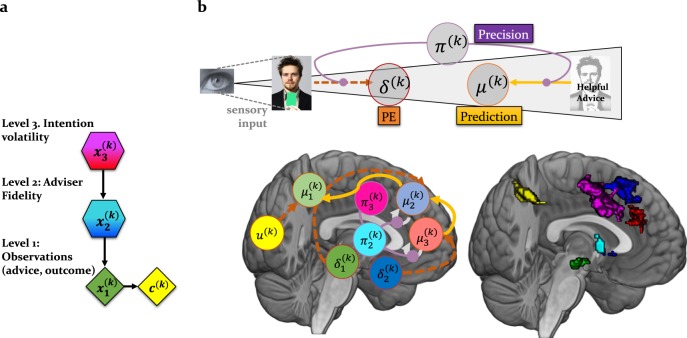
Functional anatomy of social inference: this schematic is an approximation of a neural process model of social inference. The neural signatures of the computational quantities are based on the previous, reproduced fMRI results [62]. **a** The hidden states that the agent infers on are arranged in a hierarchy as proposed by the HGF. In this graphical notation, diamonds represent quantities that change in time (i.e., that carry a time/trial index *k*). Hexagons, like diamonds, represent quantities that change in time, but additionally depend on the previous state in a Markovian fashion. From top to bottom, *x*_3_ represents the current volatility of the adviser’s intentions, *x*_2_ the adviser’s fidelity or tendency to give helpful advice, and *x*_1_ represents the accuracy of the current observation (advice or cue). **b** The inferred states are represented by circles. Thus, based on the empirical findings, we propose the following theoretical neural model of social inference: Cue-related PEs update predictions about the visual outcome and are conveyed via projections from lingual gyrus to posterior parietal cortex, whereas advice PEs, which update the advice accuracy, are passed from low-level regions (including the VTA) to higher-level “theory of mind” regions, i.e., for example, dorsomedial PFC. High-level volatility PEs are further transmitted via the cholinergic septum to cingulate regions. The precisions (advice and volatility) modulate the impact of PEs on medial PFC activity

In the context of learning about intentions, different hypotheses about how participants took decisions (i.e., going with or against the advice) were formalised in terms of a model space, which comprised different models of learning and belief-to-action mapping, including reinforcement learning models, which were formally compared [66]. The model, which best captured behaviour in this social learning task across multiple data sets [62, 63, 65], was the hierarchical Gaussian filter (HGF) [28, 29], which emphasised the role of hierarchical precision-weighted PEs in belief updating (Fig. 2b). Irrespective of participant–adviser assignment, but specific to the social task, we observed the same winning model, which assumed hierarchical learning about the advice and adviser volatility as the mechanism for mapping beliefs to decisions [65].

In previous studies, the inferred adviser fidelity and volatility of intentions, - estimated with the HGF, - reflected participants’ overtly expressed beliefs about the adviser’s intentions at different times during the task. Furthermore, the learning parameters describing each individual’s belief updates predicted participants’ ratings of their own perspective-taking tendencies, suggesting that the model captures key aspects of social cognition [62, 65].

According to this model, surprising advice outcomes have a greater impact on the agent’s internal representation (should have more influence on the belief update) when the sensory precision from the level below (i.e., $\hat \pi _{i - 1}^{\left( k \right)}$) is high. For example, a participant may have regarded unexpected misleading advice as evidence that the adviser has changed the strategy, thus adapting his/her beliefs about the adviser’s intentions and decisions to follow the advice. However, if one has a strong prior belief that the adviser’s intentions are to mislead, then the belief precision (i.e., $\pi _i^{\left( k \right)}$) is high and contrary evidence (i.e., surprising helpful advice) will be ignored.

In summary, our proposal suggests that persecutory delusions can be understood as an imbalance between sensory and belief precision. Sensory precision augments the impact of social PEs on beliefs about fidelity, and likely marks the early stages of psychosis, whereas belief precision has the opposite effect on belief updates and may reflect the consolidation of delusions. This is because belief precision refers to the confidence in one’s model of intentions, which functions to “explain away” instances of incorrect advice.

One could appreciate the distinct impact of the sensory compared with the belief precision on the belief-updating process with simulations (see Supplementary Figure 1) and by considering the following intuitive example: imagine that you buy bread from your local baker every morning. Every time, he/she offers you one of the two types of bread that is freshest that day. One day, you get very ill after eating the loaf of bread recommended to you, implying an overly high precision at the sensory level. The next day, the baker recommends you confidently the same bread. You conclude he/she must have no clue about bread, and choose the other option (i.e., opposite of his/her advice). This reflects a process of “explaining away” PEs, by adopting a new prediction. It turns out that the other bread has an intense, pungent smell (referring to the aberrant salience of sensory inputs). This leads you to believe that the baker is purposely trying to poison you with bad bread, and even when he/she recommends a “good” bread, that others in the store also buy, it further confirms your prediction that it is part of an elaborate plan to coax you to trust him/her again. This reflects the adoption of false and precise high-level beliefs, which can fully explain any instance of aberrant PEs. The aberrantly high precision on the higher-level beliefs is an adjustment in order to down-weigh the precision with respect to the sensory input (i.e., unexpected bad bread).

## Functional anatomy of social inference

The computational quantities entering the belief-updating process have been associated with neuromodulatory systems specifically implied in the pathophysiology of psychosis (for reviews, see ref. [33, 58, 67]).

In the context of social learning, we demonstrated a dichotomy between low- and high-level precision-weighted PEs as they were related to dopaminergic and cholinergic systems [62]. Whereas low-level precision-weighted PEs about advice were represented in the dopaminergic midbrain and dopaminoceptive regions such as the anterior cingulate cortex, medial, and dorsolateral PFC, high-level precision-weighted PEs about the adviser’s intentions were represented in the cholinergic septum and one specific targeted projection, the dorsal anterior cingulate cortex. Consistent results reproduced in two fMRI studies reflect fundamental neural computational architectures underlying social inference (Fig. 2).

Not surprisingly, as social inference is particularly impaired in individuals at risk for psychosis [68], the regions which encode these particular computational quantities include dopaminergic nuclei and dopaminoceptive areas, such as the striatum, shown to be affected in those at risk of developing psychosis [69, 70] and in those who later transitioned to schizophrenia [71].

## Clinical predictions afforded by computational model

As persecutory delusions predominate in major psychotic disorders and contribute to symptom severity, computational models that explain their formation and persistence may shed light onto the neural mechanisms that mark the different stages of psychosis.

**Fig. 3:**
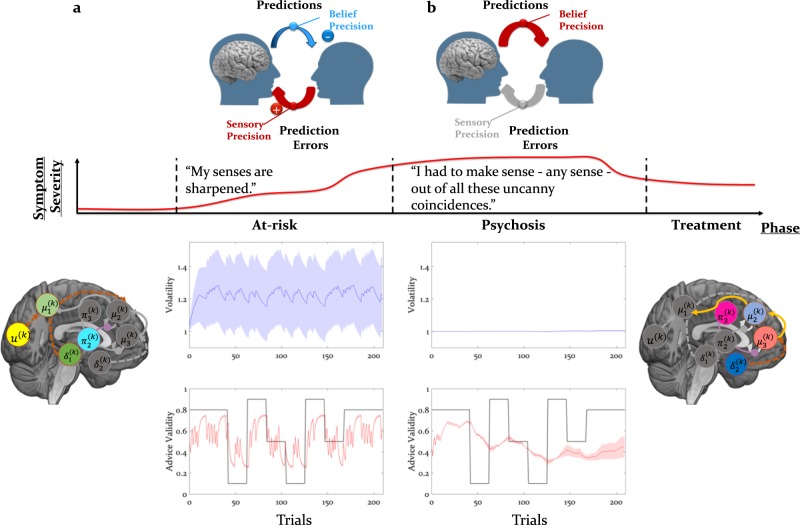
Model predictions: beliefs and neural responses: considering the psychosis spectrum timeline, one can make specific hypotheses about the parameters that could mark each stage by referring to the equation in Fig. 1 and the functional anatomy of social inference (Figs. 1 and 2) using simulations. **a** In the early, prodromal stage of increased aberrant salience, we predict an increased representation of sensory precision or $\hat \pi _{i - 1}^{\left( k \right)}$ during the social learning task. Neurally, this may be expressed as enhanced low-level PEs and thus enhanced connectivity between dopaminergic and sensory to parietal and frontal regions; **b** In the later stages, when persecutory delusions are present, we predict an enhancement of the belief precision or $\pi _i^{\left( k \right)}$ during the social learning task. At the level of the hierarchical Bayesian model, this would be associated with reduced estimated volatility, tonic learning rate, and a more negative prior estimate about the adviser’s fidelity. Neurally, this may be expressed as increased high-level precision and PEs and thus increased connectivity strength from the medial prefrontal regions to cingulate areas

In the context of social learning, we predict that the high-risk state is defined by an imbalance between the precision of beliefs at low compared with high levels of the processing hierarchy, as suggested by recent studies of perceptual inference in relation to delusions [72, 73]. Thus, the precision associated with advice PEs will likely be larger compared with the precision of the prediction about intentions, leading to a high learning rate and a reduced ability to form a cohesive model of the adviser’s intentions, which could be predicted using simulations (Fig. 3a).

Based on neuroimaging results in the healthy population [62, 63] and recent studies of aberrant salience in the at-risk population [22, 23, 74], several hypotheses about pathophysiology can be put forward, which could be falsified in future studies: first, the early prodromal stage of psychosis may be marked by an increased low-level (sensory) precision. Consistent with previous connectivity studies [75–77], this would be translated into enhanced bottom–up connectivity from dopaminergic regions to key brain regions involved in the representation of social (advice) PEs, including the temporal–parietal junction and dorsomedial prefrontal cortices [61, 62]. Thus, parameters that will likely predict transition to frank psychosis include learning parameters that determine the dynamics of precision-weighted PEs (see ref. [65]) as well as the connectivity strengths of bottom–up connections from dopaminergic to parietal and prefrontal cortices (Fig. 3a).

In the later stages of psychosis, the presence of delusions might reflect a compensatory response to the aforementioned deficiencies of hierarchical inference. Thus, in individuals who exhibit persecutory delusions, we predict an increased representation of high-level belief precision about the other’s intentions (Fig. 3b). This notion of rigid high-level priors leads to several experimentally testable predictions: at the behavioural level, this will likely be reflected as a reduced estimate of volatility. At the neural level, this will be expressed as either (i) a reduction in bottom–up connectivity from dopaminergic regions to parietal and medial prefrontal cortices, reflecting the suppression of incoming PE signals, or (ii) enhancement of top–down connectivity from cingulate to medial prefrontal and to parietal regions, reflecting an enhancement of the precision of predictions about intentions, or (iii) a combination of both (Fig. 3b). Although reduction in functional connectivity has featured prominently in the literature, in particular between temporal and prefrontal regions [78, 79], enhanced connectivity was also reported [80, 81].

An alternative hypothesis is that the pathophysiology underlying persecutory delusions is unrelated to precision, but instead to social PEs. Accordingly, individuals with persecutory delusions regard the adviser as purposely misleading, and therefore place greater weight on negative advice PEs. At the neural level, this would be expressed as biased predictions and enhanced PE signals for misleading advice.

## Testable designs

We propose two experimental designs to test our hypotheses: (1) Individuals with high-risk of developing psychosis and patients with persecutory delusions could be compared in a cross-sectional design. However, although generative modelling approaches may be useful for identifying inference and neurobiological processes leading to psychosis, validation studies are needed to determine their clinical utility. Regardless of how well a model may capture a putative pathophysiology, it needs to support differential diagnosis or prognosis, for example, by predicting transition to psychosis or treatment response with sufficient accuracy and in individual patients. (2) This can only be tested in prospective studies where CHR individuals and FEP patients who receive first-line treatment are assessed at multiple time points and model parameters are used to predict transition to psychosis or treatment response, respectively.

From previous studies of aberrant learning in psychosis, it is unclear whether alterations in social inference are specifically required to explain persecutory delusions. In fact, alterations in higher-level inferential processes that are not necessarily specific to social contexts may affect processing of socially relevant information and produce delusions. To address this question, a control task that removes the aspect of intentionality may be needed. We have previously included such a control task [65] with blindfolded advisers who selected their advice from pre-defined card decks, thus eliminating the effect of intentionality, and demonstrated that the computational model proposed here, which assumes hierarchical learning about the advice and volatility of the adviser’s intentions as the mechanism for mapping beliefs to decisions specifically captured the intentionality behind the advice [65]. In terms of more broadly distinguishing between mechanisms of abnormal plasticity linked to psychosis, additional perceptual learning tasks that tap into different mechanisms, including intact NMDA receptor signalling, such as, for example, the auditory mismatch negativity task [82] may also be needed.

## Conclusion and future directions

Mechanistically interpretable generative models like the ones outlined here allow for model comparison and testing of competing hypotheses as well as inference on disease mechanisms in individual patients at different stages of psychosis. Furthermore, the computational quantities derived from the model—such as the low- and high-level, precision-weighted PEs—could be associated with distinct neuromodulatory systems, dopaminergic and cholinergic [62], respectively, which are ultimately the targets of pharmacological treatment in psychosis. Future studies in subclinical and clinical populations will examine the usefulness of this approach for predicting transition to psychosis or treatment response in individual patients.

## Supplementary information

Supplementary Information

Supplementary Figure 1

Supplementary Figure 2

Supplementary Figure 3

## Data Availability

The routines for all simulations used here are available as Matlab code: https://gitlab.ethz.ch/compi_sim. The simulations in this paper can be reproduced by following the instructions of the ReadMe file.
