# Supplementary material for: Models of persecutory delusions: a mechanistic insight into the early stages of psychosis
Source: Mol Psychiatry. 2019 May 10;24(9):1258–67. doi: 10.1038/s41380-019-0427-z (PMC6756090; doi:10.1038/s41380-019-0427-z)
Supplement: Supplementary file 1 — Supplementary Information [file 41380_2019_427_MOESM1_ESM.docx]

# Supplementary Information

## Generative Models

The computational framework we propose here is a generative one, which assumes a specific mechanism for linking decisions of distrust to precision of beliefs about intentions. We opted for a generative approach instead of a discriminative one, to uncover the underlying mechanisms of formation and persistence of persecutory delusions: We were particularly interested in the inferential and neural causes leading to aberrant social decisions. Importantly, we hypothesize based on previous findings ^1^ that the neural processes underlying aberrant precision-weighted prediction error (PE) learning in the social context offer insight into pathophysiological processes that could be the target of treatment. This approach allows us to make predictions about treatment response in individual patients (Figure 1).


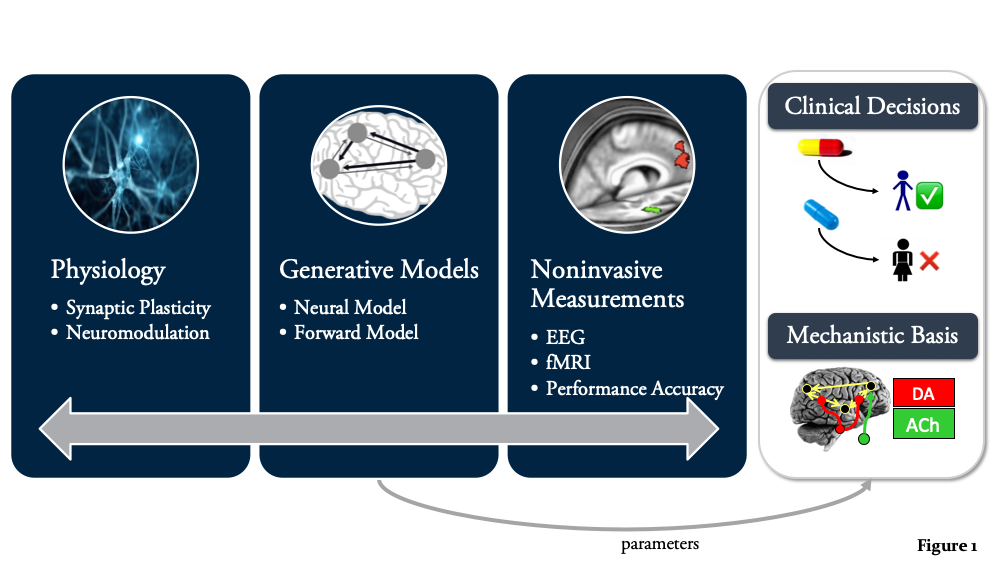
**Figure 1 | Generative Models of Behaviour and Neuroimaging Data**: Generative models represent the joint probability of data and model parameters and allow us to infer on physiological mechanisms from noninvasive measurements. *Inference* refers to the application of mathematics to draw conclusions in the presence of uncertainty. This approach is *mechanistic* in the sense that it allows to identify causes of learning (e.g., behavioural – PE and precision - or neuronal - synaptic plasticity and neuromodulation). In the context of psychosis, parameters capturing key aspects of pathology (e.g., disruptions of the dopamine system) can be used to make clinical predictions such as treatment response, in order to inform clinical decisions, such as whether a given individual should be administered medication with a principally dopaminergic action.

## Hierarchical Gaussian Filter: Computational Model of Persecutory Delusions

The computational framework we propose here is a meta-Bayesian one based on the “Observing the observer” approach ^2,3^, which assumes that agents infer on a hierarchy of hidden states $x_{1}^{(k)},x_{2}^{(k)},\ldots, x_{n}^{\left( k \right)}$ that cause the inputs they experience on each trial *k*. This assumes a generative model that causes the social inputs, i.e., trialwise instances of helpful or incorrect advice (Figure 2a) as well as a generative model that the agent uses to infer on these causes (Figure 2b). Decisions to go with or against the advice are thus based on the latter, the inferred, hierarchically-organized quantities.


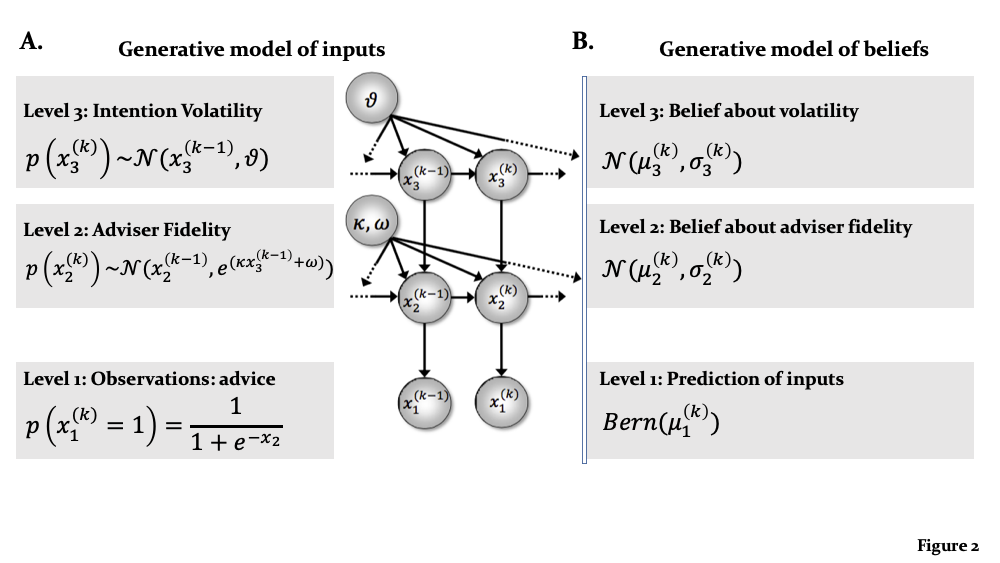
**Figure 2 | Hierarchical Gaussian Filter: Computational Model of Persecutory Delusions.** The computational model framework we propose assumes that agents infer on a hierarchy of hidden states $x_{1}^{(k)},x_{2}^{(k)},\ldots, x_{n}^{\left( k \right)}$ that cause the inputs they experience on each trial *k*. This assumes a generative model that causes the social inputs (i.e., model of the adviser’s strategy) (A). In this graphical notation, the variables with horizontal arrows change in time and additionally depend on the previous state in time in a Markovian fashion. x_1_ represents the accuracy of the current piece of advice, x_2_ the adviser’s fidelity or tendency to give helpful advice and x_3_ the current volatility of the adviser’s intentions. Parameter κ determines how strongly x_2_ and x_3_ are coupled, $\omega_{2}$ determines the tonic component of the log-volatility, and $\vartheta$ represents the meta-volatility and determines the variance of x_3_. (B) Inversion of the generative model of advice in (A) (i.e., model of how the player learns about the adviser’s strategy) is the application of Bayes’ rule. “Beliefs” refer to posterior probability distributions as described by their sufficient statistics. Assuming Gaussian distributions, these agent-specific beliefs are denoted by their summary statistics, i.e., $\mu$ (mean) and $\sigma$ (variance/uncertainty).

### Generative model of social inputs

In the current context, the model assumes that advice accuracy varies as a function of hierarchically-coupled hidden states $x_{1}^{(k)},x_{2}^{(k)},\ldots, x_{n}^{\left( k \right)}$. They evolve in time by performing Gaussian random walks. At any given level, the step size is controlled by the state of the next-higher level.

Starting from the bottom of the hierarchy, state $x_{1}$ represents binary (0 or 1) variables or advice accuracy (1 for accurate, 0 for inaccurate). Thus, the probability advice is accurate is denoted by Equation 1, and depends on the next higher (unbounded) state $x_{2}$ via the logistic sigmoid transformation s(∙) (Equation 2).

$p\left( x_{1} | x_{2} \right)={s\left( x_{2} \right)}^{x_{1}}\left( 1-s(x_{2}) \right)^{1-x_{1}}=\mathrm{Bernoulli} (x_{1};s\left( x_{2} \right))$ (1)

where

$s\left( x \right)≝\frac{1}{1+\exp\left( -x \right)}.$ (2)

States higher than $x_{1}$ are continuous. They represent the adviser fidelity and the rate of change of the advisor’s intentions:

At the next higher level, state $x_{2}$ or the adviser’s fidelity denotes the adviser’s tendency to deliver accurate advice (i.e., the adviser’s current degree of helpfulness). The variance or the step size of $x_{2}$ depends on the level above, state $x_{3}$ (Equation 3).

$p\left( x_{2}^{(k)} | x_{2}^{(k-1)}, x_{3}^{(k)}, \kappa,\omega\right)\mathcal{=N}\left( x_{2}^{(k)};x_{2}^{(k-1)},\exp\left( \kappa x_{3}^{(k)}+\omega\right) \right)$ (3)

State $x_{3}$ or the rate of change of the adviser’s intentions represents the (log) volatility of the adviser’s fidelity (Equation 4).

$p\left( x_{3}^{(k)} | x_{3}^{(k-1)}, \vartheta\right)\mathcal{=N}\left( x_{3}^{(k)};x_{3}^{(k-1)},\vartheta\right)$ (4)

The evolution of these states in time depends on three parameters: (i) $\kappa$ determines the degree to which $x_{2}$ (adviser fidelity) is coupled to $x_{3}$ (rate of change of intentions), (ii) $\omega$ represents the constant (tonic) component of the log-volatility of the fidelity, and (iii) $\vartheta$ determines how quickly the rate of change evolves in time (i.e., the step size of the Gaussian random walk performed by $x_{3}$).

$p\left( x_{1}^{(k)}, x_{2}^{(k)}, x_{3}^{(k)}, x_{2}^{(k-1)}, x_{3}^{(k-1)}|\kappa,\omega, \vartheta\right)=p\left( x_{1}^{(k)} | x_{2}^{(k)} \right)p\left( x_{2}^{(k)} \right|x_{2}^{(k-1)},x_{3}^{(k)}, \kappa, \omega)p\left( x_{3} \right|x_{3}^{(k-1)},\vartheta)p(x_{2}^{(k-1)},x_{3}^{(k-1)})$ (5)

This constitutes the *generative model* of the outcomes observed by the agent (Figure 2a).

### Inversion of the model: The observer’s point of view

In accordance with Bayes’ rule, we assumed that participants who make inferences on advice outcomes, form posterior beliefs over the hidden states (i.e., advice-outcome congruency) based on the inputs they observe. Model inversion is the application of Bayes’ rule to a generative model such as the one described above. This leads to a *recognition* or *perceptual model*, which describes subjects’ beliefs about hidden states.

The update equations are then derived by variational calculus and provide approximately Bayes-optimal rules for trial-by-trial updating of an agent’s beliefs, given this agent’s particular set of parameter values ( for details, see ^4,5^). “Belief” refers to a posterior probability distribution as described by its sufficient statistics: Assuming Gaussian distributions, agent-specific beliefs are denoted by their summary statistics, i.e., $\mu$ (mean) and $\sigma$ (variance/uncertainty) or its inverse $\pi=1/\sigma$ (precision/certainty). Predictions or prior beliefs about the hidden states (before observing the outcome) are denoted with a hat symbol (e.g.,$\hat{\mu}$).

On trial *k*, an observed trial outcome *u* indicates that advice was either accurate $(u^{(k)}=1)$ or inaccurate ${(u}^{(k)}=0)$. At the bottom level, there is complete correspondence between observation $u^{(k)}$ and posterior belief $\mu_{1}^{(k)}$ because the advice accuracy is seen by the participant without ambiguity:

$$\mu_{1}^{(k)}=u^{(k)}=x_{1}^{(k)} (6)$$

The observed outcome $u^{(k)}$ leads to a hierarchical cascade of belief updates described by the update equations. First, it induces an advice prediction error (PE) $\delta_{1}^{(k)}$ with respect to the prediction $\hat{\mu}_{1}^{(k)}$ (the agent’s belief about the probability of the advice being correct after the previous trial; see Eq. 13):

$$\delta_{1}^{(k)}=u^{(k)}-\hat{\mu}_{1}^{(k)} (7)$$

The ensuing precision-weighted PE updates are hierarchically organized in the sense that the agent needs to use $\delta_{1}^{(k)}$ to update his/her second-level belief about the adviser’s fidelity. Since adviser fidelity or state $x_{2}^{(k)}$ is continuous, it is assumed to be Gaussian and thus represented by its sufficient statistics, $\mu_{2}^{(k)}$ (mean) and $\pi_{2}^{(k)}$ (precision, i.e., inverse variance). The update $\Delta\mu_{2}^{(k)}=\mu_{2}^{(k)}-\hat{\mu}_{2}^{(k)}$ to the prediction $\hat{\mu}_{2}^{(k)}=\mu_{2}^{(k-1)}$ is driven by $\delta_{1}$ and weighted by $\pi_{2}$:

$$\Delta\mu_{2}^{(k)}=\frac{1}{\pi_{2}^{(k)}}\delta_{1}^{(k)} (8)$$

This update in turn leads to a volatility PE, $\delta_{2}^{(k)}$, induced by $\Delta\mu_{2}^{(k)}$. At the third level, the agent’s belief about the phasic log-volatility $x_{3}^{(k)}$ of the adviser’s fidelity is represented by its sufficient statistics, $\mu_{3}^{(k)}$ and $\pi_{3}^{(k)}$, and the pattern from the second level repeats itself.

Specifically, the update $\Delta\mu_{3}^{(k)}=\mu_{3}^{(k)}-\hat{\mu}_{3}^{(k)}$ to the prediction $\hat{\mu}_{3}^{(k)}=\mu_{3}^{(k-1)}$ is driven by $\delta_{2}$ and weighted by $\pi_{3}$:

$$\Delta\mu_{3}^{(k)}\propto\frac{1}{\pi_{3}^{\left( k \right)}}\delta_{2}^{\left( k \right)} (9)$$

The complete form of the update equation at the third level is the following:

$$\Delta\mu_{3}^{(k)}=\frac{1}{2}\frac{\gamma_{2}^{(k)}}{\pi_{3}^{\left( k \right)}}\delta_{2}^{\left( k \right)} (10)$$

with the (auxiliary) expected precision

$$\gamma_{2}^{(k)}=\kappa v_{2}^{(k)}\pi_{2}^{(k)} (11)$$

and the predicted environmental uncertainty (as a function of the expected phasic log-volatility of the adviser’s fidelity, $\mu_{3}^{(k-1)}$):

$$v_{2}^{(k)}=\exp\left( \kappa\mu_{3}^{\left( k-1 \right)}+\omega\right). (12)$$

After performing these belief updates about the adviser’s fidelity and the volatility of his fidelity, the agent is able to update the probability $\hat{\mu}_{1}^{(k+1)}$ that the advice on the next trial will be correct. This corresponds to the logistic sigmoid of the current expectation of adviser fidelity:

$$\hat{\mu}_{1}^{(k+1)}=s\left( \mu_{2}^{(k)} \right)=\frac{1}{1+\exp\left( -\mu_{2}^{\left( k \right)} \right)} (13)$$

### Computational quantities altered in psychosis

In this paper, we propose that persecutory delusions can be understood in terms of enhanced belief precision about the adviser’s fidelity, leading to overly rigid beliefs about others.

Advice belief precision is given by

$$\pi_{2}^{(k)}=\hat{\pi}_{2}^{(k)}+\frac{1}{\hat{\pi}_{1}^{(k)}} (14)$$

with the precision of the prediction about advice given by

$$\hat{\pi}_{2}^{(k)}=\frac{1}{1/{\pi_{2}^{(k-1)}}+v_{2}^{(k)}}. (15)$$

Note that this depends on the predicted (i) sensory precision of the input, $\hat{\pi}_{1}^{(k)}$and (ii) environmental uncertainty, $v_{2}^{(k)}$. This equation reflects the distinct impact of lower- compared to higher-level sources of precision: An increase in sensory precision will have a dampening effect on the advice precision, thus reducing the confidence in one’s model of the adviser. On the other hand, a reduction in environmental uncertainty will increase the advice precision.

The rest of the high-level quantities depend on the advice belief precision. The higher-level volatility PE $\delta_{2}$ depends on $\pi_{2}$:

$$\delta_{2}^{(k)}=\frac{\hat{\pi}_{2}^{\left( k \right)}}{\pi_{2}^{\left( k \right)}}+(\pi_{2}^{\left( k \right)})^{2}\hat{\pi}_{2}^{\left( k \right)}\left( \Delta\mu_{2}^{(k)} \right)^{2}-1, (16)$$

and the higher-level, volatility $\pi_{3}$ depends on $\delta_{2}$:

$$\pi_{3}^{\left( k \right)}=\hat{\pi}_{3}^{(k)}+\frac{1}{2}\left( \gamma_{2}^{\left( k \right)} \right)^{2}+\left( \gamma_{2}^{\left( k \right)} \right)^{2}\delta_{2}^{(k)}-\frac{1}{2}\gamma_{2}^{\left( k \right)}\delta_{2}^{(k)}, (17)$$

with the precision of the prediction about volatility given by

$$\hat{\pi}_{3}^{(k)}=\frac{1}{1/{\pi_{3}^{(k-1)}+\vartheta}}. (18)$$

The update equations also reflect that an increase in belief precision about the adviser leads to a reduction in volatility PEs (Equation 16), and a perception that the environment is more stable. This, in turn, leads to an increase in the volatility precision or confidence in one’s model of the adviser’s intentions and how stable they are. In other words, the update equations show how enhanced advice precision maintains (abnormal) beliefs about others, a mechanism perhaps explaining why delusions persist in spite of disconfirmatory evidence.

### Patient subgroups defined by model parameters

The HGF model parameters capture the individual learning style of participants and determine the evolution of precisions over time (Figure 3). Three parameters, $\kappa$,$\omega$, and $\vartheta$, may define distinct patient subgroups:

- A large value of parameter $\kappa$, which reflects the coupling of the levels of the hierarchy, may describe individuals in an early phase of psychosis, experiencing an increase in the salience of sensory inputs (i.e., “My senses were sharpened; sights and sounds possess a keenness that I have never experienced before.” ^6^). According to Eqn. 12, 14-15, this leads to an increase in the perception of environmental uncertainty. A large value of parameter $\omega$, which reflects the tonic aspect of the log-volatility about the adviser fidelity, has a similar impact on the learning rate and the estimated precision of sensory inputs. However, whereas an increase in $\kappa$ has an impact on the phasic component of the learning rate, an increase omega impacts the tonic part. In other words, whereas an agent with high $\kappa$ values deploys hierarchical inference more (i.e., the changing advice validity has a stronger impact on the estimated volatility of intentions), an individual with high $\omega$ values is more likely to jump to conclusions, i.e., update his/her beliefs about the advice validity more given new advice PEs.
- On the other hand, a reduced value of parameter $\omega$ may describe individuals whose beliefs are resistant to change (i.e., “I had to make sense - any sense - out of all these uncanny coincidences. I did it by radically changing my conception of reality.” ^7^). According to Eqn. 12, 14-15, this contributes to an increase in the precision of the belief about the adviser’s fidelity ($\pi_{2}^{(k)}$).
- By virtue of Eqn. 18, parameter $\vartheta$ has an impact on the precision about the volatility of intentions. A large value of $\vartheta$ captures a more variable estimation of environmental volatility leading to enhanced uncertainty about the adviser’s intentions. When $\vartheta$ is low, the higher-level precision about the adviser’s changing intentions is high. This would describe an agent who is confident about his/her estimates of contextual change, and thus is sensitive to incoming prediction errors that could reveal contextual or global changes in advice validity.


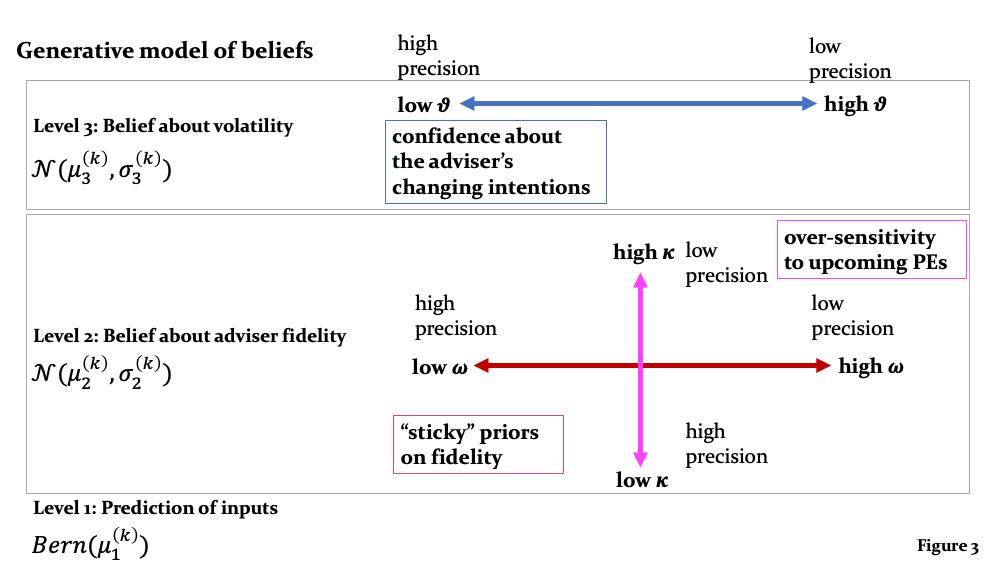


**Figure 3 | Linking HGF Parameter Values to Social Inference:** Schematic for the interpretation of the HGF parameters in relation to the generative model of beliefs about the adviser’s strategy (fidelity and intentions). The textboxes reflect the interpretation of high vs. low parameter estimates at each level of the hierarchy.

# References:

1 Diaconescu AO, Mathys C, Weber LAE, Kasper L, Mauer J, Stephan KE. Hierarchical prediction errors in midbrain and septum during social learning. *Soc Cogn Affect Neurosci* 2017; **12**: 618–34.

2 Daunizeau J, den Ouden HEM, Pessiglione M, Kiebel SJ, Stephan KE, Friston KJ. Observing the Observer (I): Meta-Bayesian Models of Learning and Decision-Making. *PLoS One* 2010; **5**. DOI:ARTN e15554 DOI 10.1371/journal.pone.0015554.

3 Daunizeau J, den Ouden HEM, Pessiglione M, Kiebel SJ, Friston KJ, Stephan KE. Observing the Observer (II): Deciding When to Decide. *PLoS One* 2010; **5**.

4 Mathys C, Daunizeau J, Friston KJ, Stephan KE. A Bayesian foundation for individual learning under uncertainty. *Front Hum Neurosci* 2011; **5**.

5 Mathys CD, Lomakina EI, Daunizeau J, *et al.* Uncertainty in perception and the Hierarchical Gaussian Filter. *Front Hum Neurosci* 2014; **8**. DOI:10.3389/fnhum.2014.00825.

6 Kapur S. Psychosis as a state of aberrant salience: a framework linking biology, phenomenology, and pharmacology in schizophrenia. *Am J Psychiatry* 2003; **160**: 13–23.

7 Clark A. Surfing Uncertainty: Prediction, Action, and the Embodied Mind. Oxford University Press, 2015.
