# Supplementary material for: Models of persecutory delusions: a mechanistic insight into the early stages of psychosis
Source: Mol Psychiatry. 2019 May 10;24(9):1258–67. doi: 10.1038/s41380-019-0427-z (PMC6756090; doi:10.1038/s41380-019-0427-z)
Supplement: Supplementary file 2 — Supplementary Figure 1 [file 41380_2019_427_MOESM2_ESM.pdf]

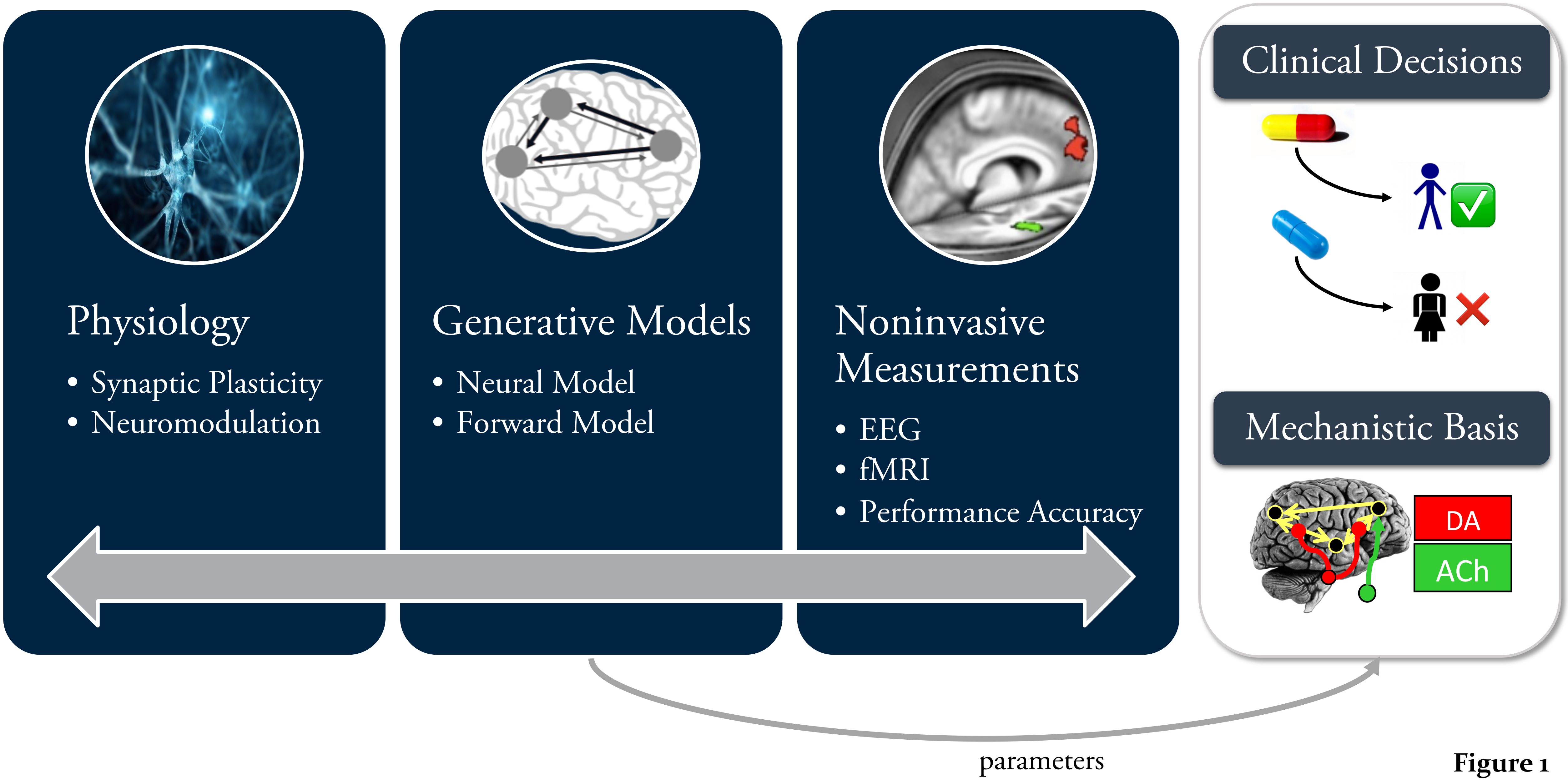
Physiology
• Synaptic Plasticity
• Neuromodulation
Generative Models
• Neural Model
• Forward Model
Noninvasive Measurements
• EEG
• fMRI
• Performance Accuracy
Clinical Decisions
Mechanistic Basis
DA
ACh
parameters

Figure 1
