# Supplementary material for: Models of persecutory delusions: a mechanistic insight into the early stages of psychosis
Source: Mol Psychiatry. 2019 May 10;24(9):1258–67. doi: 10.1038/s41380-019-0427-z (PMC6756090; doi:10.1038/s41380-019-0427-z)
Supplement: Supplementary file 3 — Supplementary Figure 2 [file 41380_2019_427_MOESM3_ESM.pdf]

A. **Generative model of inputs**

B. **Generative model of beliefs**

Level 3: Intention Volatility

$$p\left(x_3^{(k)}\right) \sim \mathcal{N}\left(x_3^{(k-1)}, \vartheta\right)$$

Level 2: Adviser Fidelity

$$p\left(x_2^{(k)}\right) \sim \mathcal{N}\left(x_2^{(k-1)}, e^{\left(\kappa x_3^{(k-1)} + \omega\right)}\right)$$

Level 1: Observations: advice

$$p\left(x_1^{(k)} = 1\right) = \frac{1}{1 + e^{-x_2}}$$

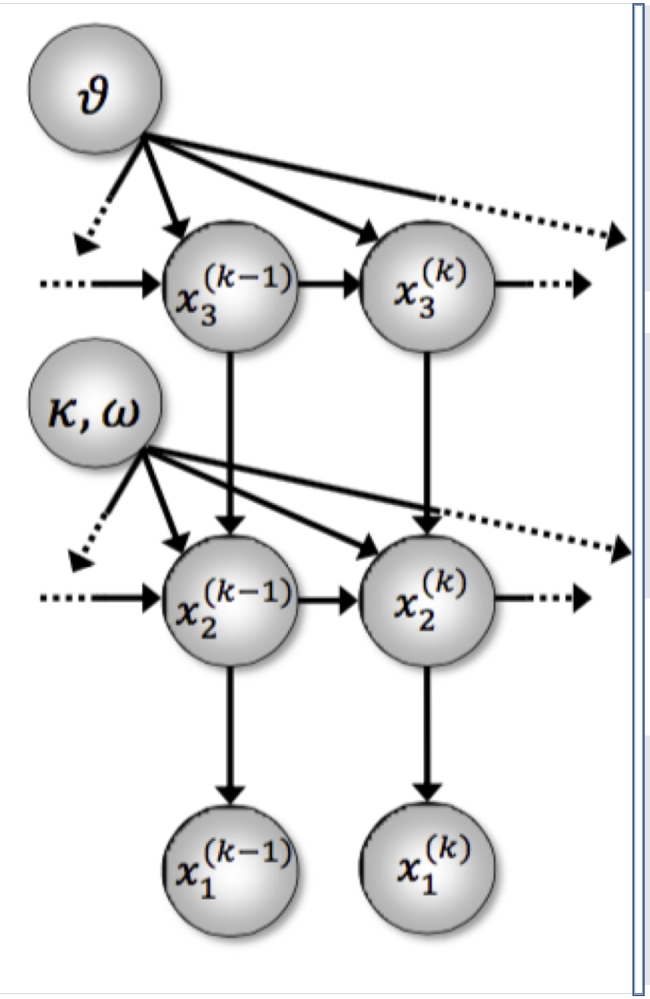

Level 3: Belief about volatility

$$\mathcal{N}\left(\mu_3^{(k)}, \sigma_3^{(k)}\right)$$

Level 2: Belief about adviser fidelity

$$\mathcal{N}\left(\mu_2^{(k)}, \sigma_2^{(k)}\right)$$

Level 1: Prediction of inputs

$$Bern\left(\mu_1^{(k)}\right)$$

Figure 2
