# Supplementary figures and images for: Models of persecutory delusions: a mechanistic insight into the early stages of psychosis
Source: Mol Psychiatry. 2019 May 10;24(9):1258–67. doi: 10.1038/s41380-019-0427-z (PMC6756090; doi:10.1038/s41380-019-0427-z)

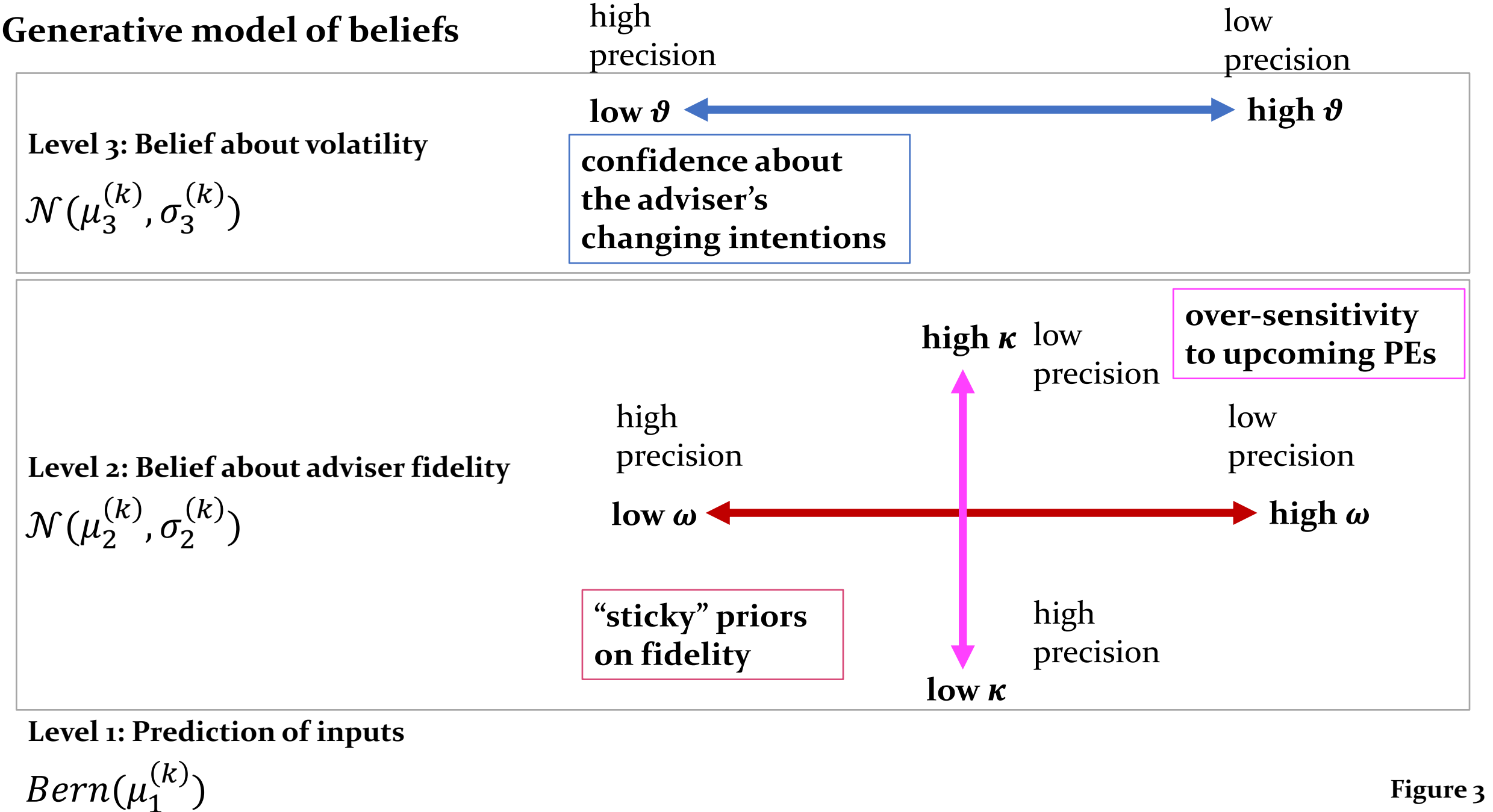

Figure 3

Supplement: Supplementary file 4 — Supplementary Figure 3 [file 41380_2019_427_MOESM4_ESM.pdf]
